# Association of SGLT2 Inhibitor Use with All-Cause Mortality Following CIED Implantation for Conduction Disease in Patients with Preserved LVEF: A Retrospective Cohort Study

**DOI:** 10.3390/jcm15145681

**Published:** 2026-07-20

**Authors:** Hidayet Ozan Arabaci, Fatih Ozkan, Zafer Guven, Muhammed Heja Gecit, Seyma Arslan, Sukru Arslan

**Affiliations:** 1 Department of Cardiology, Yuksekova State Hospital, 30300 Hakkari, Turkey; 2 Department of Cardiology, Gaziosmanpasa Training and Research Hospital, 34255 Istanbul, Turkey; 3 Department of Cardiology, Institute of Cardiology, Istanbul University-Cerrahpasa, 34098 Istanbul, Turkey; 4 Republic of Turkey Ministry of Health, 06800 Ankara, Turkey

**Keywords:** sodium–glucose cotransporter-2 inhibitors, cardiac implantable electronic devices, cardiac conduction disease, preserved left ventricular ejection fraction, all-cause mortality, bradyarrhythmia

## Abstract

**Background/Objectives**: Patients receiving a cardiac implantable electronic device (CIED) for conduction disease may remain at substantial long-term risk despite preserved systolic function at implantation. The prognostic association of sodium–glucose cotransporter-2 (SGLT2) inhibitor use in this population is uncertain. We therefore examined the relationship between early documented SGLT2 inhibitor use and subsequent all-cause mortality in patients with preserved left ventricular ejection fraction (LVEF) undergoing CIED implantation for conduction disease. **Methods**: In this retrospective single-center cohort study, we screened consecutive adults who underwent CIED implantation for conduction disease between January 2018 and June 2024. Patients with LVEF ≥ 50% and complete clinical, laboratory, electrocardiographic, and echocardiographic data were included. SGLT2 inhibitor exposure was defined as documented initiation or active use within 3 months after implantation. The primary endpoint was all-cause mortality. Associations were evaluated using multivariable Cox proportional-hazards regression and Kaplan–Meier analyses. **Results**: Among 2176 screened patients, 540 fulfilled the eligibility criteria, of whom 206 (38.1%) had documented SGLT2 inhibitor exposure. During a median observation period of 64.2 months (range, 7–90 months), 185 deaths occurred. SGLT2 inhibitor exposure was associated with a lower adjusted hazard of death (HR, 0.48; 95% CI, 0.38–0.69; *p* < 0.001). Survival also differed between exposure groups in the overall cohort (log-rank *p* = 0.006) and in patients without either heart failure or diabetes mellitus (log-rank *p* = 0.020). **Conclusions**: In patients with preserved LVEF undergoing CIED implantation for conduction disease, documented SGLT2 inhibitor use was independently associated with lower all-cause mortality. These observational findings are hypothesis-generating and warrant confirmation in prospective studies.

## 1. Introduction

Cardiac conduction disease is a major indication for cardiac implantable electronic device (CIED) implantation, particularly among older patients with substantial cardiovascular and cardiorenal comorbidity. Although a preserved left ventricular ejection fraction (LVEF) at the time of implantation may indicate preserved systolic function, it does not necessarily identify a low-risk clinical phenotype. In patients expected to require substantial ventricular pacing, conventional right ventricular pacing may contribute to electrical and mechanical dyssynchrony, adverse ventricular remodeling, and subsequent deterioration in cardiac function [1,2]. These considerations are clinically relevant in patients undergoing CIED implantation for conduction disease, including those with preserved LVEF at baseline.

Originally developed as glucose-lowering agents, sodium–glucose cotransporter-2 (SGLT2) inhibitors are now recognized as an important component of cardiorenal therapy. Large, randomized trials have shown that these agents reduce heart failure–related events across a broad range of left ventricular ejection fraction, including in patients with mildly reduced or preserved LVEF [3,4,5,6]. Based on this evidence, contemporary heart failure guidelines recommend SGLT2 inhibitors for appropriate patients with symptomatic heart failure, including those with mildly reduced or preserved ejection fraction [7]. However, whether the favorable prognostic associations observed in heart failure populations extend to patients undergoing CIED implantation for cardiac conduction disease remains uncertain.

Several mechanisms may plausibly support a potential benefit of SGLT2 inhibitor therapy in this setting, although these mechanisms have not been directly evaluated in patients with chronic ventricular pacing. Meta-analyses have reported that SGLT2 inhibitor therapy may be associated with lower risks of atrial arrhythmias and sudden cardiac death among patients with diabetes mellitus or heart failure [8,9,10]. Similarly, a recent review suggested that SGLT2 inhibitor use may reduce the risk of post-myocardial infarction arrhythmias and sudden cardiac death through potential effects on ventricular remodeling, ion-channel regulation, and myocardial energy metabolism [10]. In addition, imaging-based studies and meta-analyses have reported favorable changes in left ventricular myocardial mechanics and right ventricular function with SGLT2 inhibitor therapy [11,12]. Together, these observations provide a biologically plausible rationale for evaluating the prognostic relevance of SGLT2 inhibitor use in patients with device-treated conduction disease.

Evidence specifically addressing CIED-treated populations remains limited. In a cohort of patients with implanted cardiac devices, SGLT2 inhibitor use was associated with lower atrial tachyarrhythmia burden and reduced all-cause mortality [13]. More recently, a population-based study of conventional pacemaker recipients with atrioventricular block reported associations between SGLT2 inhibitor use and lower risks of all-cause mortality and heart failure hospitalization after implantation [14]. Nevertheless, data remain sparse in patients whose primary indication for CIED implantation is cardiac conduction disease and who have preserved LVEF at baseline.

Accordingly, the present study evaluated the association between documented SGLT2 inhibitor use and all-cause mortality in patients with preserved LVEF undergoing CIED implantation for cardiac conduction disease. Exploratory analyses also assessed whether this association was observed in clinically relevant subgroups, including patients without heart failure, diabetes mellitus, or both conditions.

## 2. Materials and Methods

### 2.1. Study Population

**Figure 1:**
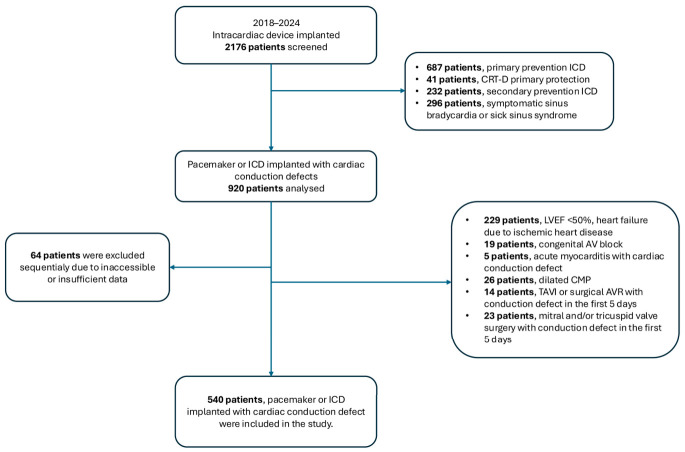
The Flowchart of the Study. AV: atrioventricular; AVR: aortic valve replacement; CMP: cardiomyopathy; CRT-D: cardiac resynchronization therapy-defibrillator; ICD: implantable cardioverter-defibrillator; TAVI: transcatheter aortic valve implantation.

This was a single-center, retrospective observational cohort study; therefore, the analyses were designed to evaluate associations rather than establish causal or therapeutic effects. Using the institutional electrophysiology database, we identified adults who underwent CIED implantation for a cardiac conduction disorder from 1 January 2018 through 30 June 2024. Patients were eligible when baseline LVEF was ≥50% and the records contained adequate preimplant clinical, laboratory, electrocardiographic, and echocardiographic information. Exclusion criteria were applied sequentially, and each patient was counted only once in the flowchart. Patients undergoing ICD or CRT-D implantation for primary or secondary prevention were excluded at screening; however, patients whose primary implantation indication was cardiac conduction disease and who additionally required an ICD for secondary prevention were eligible. Patients with sick sinus syndrome or symptomatic sinus bradycardia, ischemic or nonischemic dilated cardiomyopathy with LVEF < 50%, congenital AV block, acute myocarditis-related conduction defects, valvular intervention-related conduction defects, or missing or inaccessible data were excluded. Sequential application of the eligibility criteria reduced the initial 2176 records to an analytic cohort of 540 patients, as detailed in Figure 1.

The institutional source population partially overlaps with a previously published cohort used to investigate the prognostic value of the T-AMYLO score and cardiac amyloidosis red flags [15]. The present analysis addresses a distinct research question by restricting eligibility to patients who underwent device implantation between 2018 and 2024, had an LVEF ≥ 50%, and had documented SGLT2 inhibitor exposure assessed in relation to all-cause mortality. Of the 540 patients included in the present study, 198 (36.7%) had also been included in the previous report.

### 2.2. Definitions and Device Implantation Indications

Implantation indications were classified into three categories: AV block, non-AV conduction disease, and permanent atrial fibrillation (AF) with a slow ventricular response. The AV block group comprised symptomatic 2:1 AV block, advanced AV block with conduction ratios such as 3:1 or 4:1, and complete AV block. Non-AV conduction disease was defined as symptomatic bradycardia associated with bifascicular or trifascicular conduction abnormalities. For the purposes of this study, bifascicular disease was operationally defined as left anterior fascicular block accompanied by first-degree AV block, complete right bundle branch block (RBBB) combined with left anterior fascicular block, or complete RBBB accompanied by first-degree AV block. Trifascicular disease was defined as complete left bundle branch block (LBBB) with first-degree AV block or incomplete LBBB occurring together with complete RBBB and first-degree AV block. AF with slow ventricular response was defined as permanent AF accompanied by symptomatic bradyarrhythmia and a ventricular rate below 50 beats/min.

All implantation indications were determined by the Arrhythmia and Electrophysiology Clinic in accordance with contemporary European Society of Cardiology and American Heart Association guidelines on bradyarrhythmias and device implantation [16,17].

### 2.3. Data Collection and Clinical Assessment

Demographic characteristics, clinical history, medication use, laboratory findings, electrocardiographic and echocardiographic data, and physical examination findings were obtained from institutional electronic medical records and the Arrhythmia and Electrophysiology Clinic database. SGLT2 inhibitor exposure was defined according to documented treatment use within the first three months following CIED implantation, based on the available national prescription records and institutional medical records. Owing to the retrospective nature of the study, the indication for treatment, the specific SGLT2 inhibitor and dose used, adherence, persistence, discontinuation, and subsequent treatment modifications could not be consistently ascertained. Renal impairment was categorized as chronic kidney disease when the estimated GFR was below 60 mL/min/1.73 m^2^. Coronary artery disease required either angiographic stenosis of at least 50% or previous coronary revascularization. Heart failure was identified from a documented clinical diagnosis supported by compatible symptoms or signs, New York Heart Association functional class, natriuretic peptide levels when available, and echocardiographic structural or functional abnormalities consistent with elevated filling pressures in the presence of LVEF ≥ 50%. Formal HFA-PEFF or H2FPEF scores were not systematically calculated. Data on ventricular pacing burden, paced QRS duration, conduction system pacing, remote monitoring, and longitudinal device interrogation parameters were not systematically available.

The study was conducted in accordance with the Declaration of Helsinki and was approved by the Institutional Review Board of Istanbul University-Cerrahpasa (E-74555795-050.04-1308927, 15 May 2025).

### 2.4. Electrocardiographic and Echocardiographic Evaluation

Baseline rhythm, atrioventricular conduction, and ventricular depolarization were characterized using electrocardiograms recorded before device implantation. The assessed variables comprised PR and QRS intervals, bundle branch and atrioventricular block patterns, and the heart rate-corrected QT interval, which was derived using Bazett’s formula. Interval measurements were reported in milliseconds [18].

Echocardiographic data were extracted from transthoracic studies performed either before implantation or during the index hospitalization. The analysis included interventricular septal and posterior wall thicknesses, left ventricular end-diastolic diameter, left ventricular ejection fraction, left atrial diameter, diastolic function, tricuspid annular plane systolic excursion, and estimated systolic pulmonary artery pressure [19]. Serial echocardiographic assessments after device implantation were not consistently available and were therefore not included in the analysis.

### 2.5. Outcome and Follow-Up

The primary endpoint was all-cause mortality. Mortality data were obtained from the national patient registry database and institutional death notification records. Follow-up was calculated from the date of CIED implantation to death or the last available follow-up, whichever occurred first.

### 2.6. Statistical Analysis

All statistical analyses were performed using SPSS version 24.0 (SPSS Inc., Chicago, IL, USA). Continuous variables are presented as mean ± standard deviation or median (minimum–maximum), according to distribution, and categorical variables as counts and percentages. Between-group comparisons were performed using Student’s *t*-test or the Mann–Whitney U test for continuous variables and Pearson’s chi-square or Fisher’s exact test for categorical variables, as appropriate.

Kaplan–Meier methods were used to estimate survival probability, and groups were compared with the log-rank test. Survival plots display follow-up in months, group sample sizes, event counts, censoring marks, 95% confidence intervals, and number-at-risk tables. Multivariable Cox proportional hazards regression was used to evaluate variables independently associated with all-cause mortality. Variables with *p* < 0.10 in univariable analyses or established clinical relevance were considered for the multivariable model. Hazard ratios (HRs) with 95% confidence intervals (CIs) are reported. Potential multicollinearity was assessed using variance inflation factors.

Exploratory subgroup analyses were performed according to diabetes mellitus, chronic kidney disease, heart failure, and the combined absence of heart failure and diabetes mellitus. Within each subgroup, the association between SGLT2 inhibitor use and all-cause mortality was evaluated using bivariate analyses and Kaplan–Meier survival analysis. All analyses were two-sided, and *p* < 0.05 was considered statistically significant. Given the retrospective observational design, results are reported as associations and should not be interpreted as causal treatment effects.

## 3. Results

A total of 540 patients were included in the final analysis. The mean age was 76.9 years, and 292 patients (54.1%) were women. Heart failure was present in 114 patients (21.1%), diabetes mellitus in 203 (37.6%), chronic kidney disease in 161 (29.8%), coronary artery disease in 150 (27.8%), and AF in 227 (42.0%). The implantation indication was AV block in 340 patients (63.0%), non-AV conduction disease in 62 (11.5%), and AF with a slow ventricular response in 138 (25.6%). A VR pacemaker was implanted in 182 patients (33.7%), a DR pacemaker in 296 (54.8%), and an ICD in 62 (11.5%). SGLT2 inhibitor use was documented in 206 patients (38.1%). The median follow-up duration was 64.2 months (range, 7–90 months). No deaths occurred within the first 3 months after CIED implantation. During follow-up, all-cause mortality occurred in 185 patients (34.3%).

Clinical and demographic characteristics according to all-cause mortality are presented in Table 1. Patients who died were older than survivors (median, 86 vs. 76 years; *p* < 0.001), whereas sex and implantation indications were similarly distributed between groups (*p* = 0.581 and *p* = 0.770, respectively). Device types differed between groups (*p* < 0.001), with VR pacemakers and VR-ICDs more frequent among patients who died. Heart failure was more prevalent among patients with all-cause mortality than among survivors (28.1% vs. 17.5%; *p* = 0.006).

Diabetes mellitus, hypertension, prior stroke, and chronic kidney disease were more common among patients who died during follow-up, whereas coronary artery disease, atrial fibrillation, and smoking status were similarly distributed between groups (Table 1). Syncope or presyncope was less frequent among patients who died (69.2% vs. 77.5%; *p* = 0.016). SGLT2 inhibitor use was more frequent among survivors (42.5% vs. 29.7%; *p* = 0.004), whereas amiodarone, digoxin, mineralocorticoid receptor antagonist, diuretic, and oral anticoagulant use was more frequent among patients who died.

Clinical and demographic characteristics according to SGLT2 inhibitor use are presented in Table 2. Age, sex, implantation indications, and device types were comparable between users and non-users. Heart failure (23.3% vs. 19.8%; *p* = 0.327) and AF (46.1% vs. 39.5%; *p* = 0.132) were numerically more frequent among users, although neither difference was statistically significant. In contrast, diabetes mellitus (45.1% vs. 32.9%; *p* = 0.004), hypertension (89.3% vs. 79.9%; *p* = 0.004), coronary artery disease (36.2% vs. 22.5%; *p* < 0.001), and chronic kidney disease (35.4% vs. 26.3%; *p* = 0.022) were significantly more common among SGLT2 inhibitor users.

Electrocardiographic and echocardiographic findings are summarized in Table 3. PR interval duration and the prevalence of complete AV block were similar between groups. QRS duration and QTc interval were longer among patients with all-cause mortality (both *p* < 0.05).

LVEF and TAPSE were similar between groups (*p* = 0.389 and *p* = 0.349, respectively). In contrast, left atrial diameter, interventricular septal thickness, and systolic pulmonary artery pressure were higher among patients with all-cause mortality (Table 3).

Laboratory and biochemical findings are shown in Table 4. Hemoglobin and hematocrit levels were lower among patients with all-cause mortality, whereas platelet and total white blood cell counts were similar. Neutrophil count, C-reactive protein, creatinine, troponin T, and NT-proBNP levels were higher in patients who died during follow-up, while GFR was lower.

Patients with all-cause mortality had higher serum creatinine levels and lower GFR than survivors (0.9 vs. 1.1 mg/dL and 67.1 vs. 51.5 mL/min, respectively; both *p* < 0.001). Troponin T and NT-proBNP levels were also higher in patients who died during follow-up (0.012 vs. 0.016 ng/mL and 167.7 vs. 275.0 pg/mL, respectively; both *p* = 0.001).

Table 5 presents the multivariable Cox proportional hazards model for all-cause mortality. After adjustment for demographic characteristics, comorbidities, electrocardiographic and echocardiographic parameters, renal function, hematologic indices, and log-transformed NT-proBNP, SGLT2 inhibitor use remained independently associated with lower mortality (HR, 0.48; 95% CI, 0.38–0.69; *p* < 0.001). Other independent predictors were older age (HR per year, 1.05; 95% CI, 1.03–1.07; *p* < 0.001), complete LBBB (HR, 1.57; 95% CI, 1.29–2.24; *p* = 0.001), higher log-transformed NT-proBNP (HR, 1.26; 95% CI, 1.12–1.41; *p* = 0.003), lower GFR (HR per mL/min, 0.98; 95% CI, 0.97–0.99; *p* = 0.004), and greater interventricular septal thickness (HR per mm, 1.18; 95% CI, 1.03–1.42; *p* = 0.022). AF (HR, 1.41; 95% CI, 0.99–1.91; *p* = 0.061) and complete AV block (HR, 1.32; 95% CI, 0.97–1.80; *p* = 0.077) showed nonsignificant associations toward higher mortality. All variance inflation factor values were below 3.0, indicating no significant multicollinearity.

**Figure 2:**
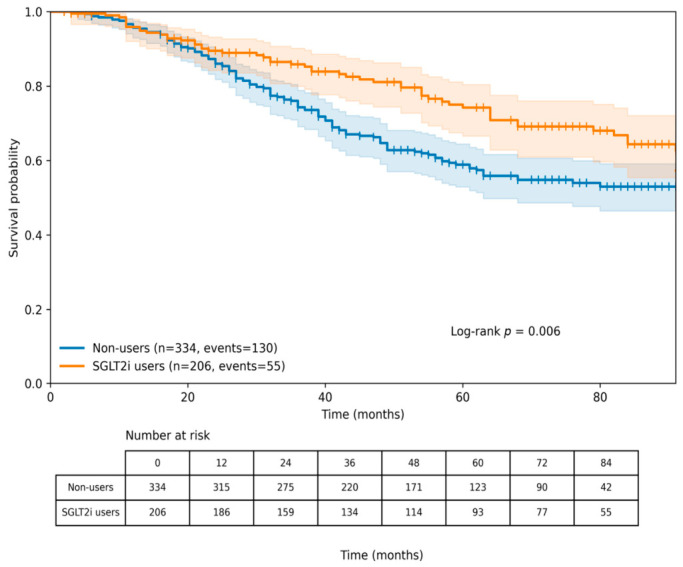
Kaplan–Meier Curves for Overall Survival According to SGLT2 Inhibitor Use in the Overall Cohort. SGLT2 inhibitor users had significantly higher survival probability than non-users (log-rank *p* = 0.006). Shaded areas represent 95% confidence intervals, tick marks indicate censored observations, and numbers at risk are shown below the plot. The endpoint was all-cause mortality. SGLT2i: sodium–glucose cotransporter-2 inhibitor.

Kaplan–Meier analysis showed significantly higher survival probability among SGLT2 inhibitor users than non-users in the overall cohort (log-rank *p* = 0.006; Figure 2). During follow-up, all-cause mortality occurred in 55 of 206 SGLT2 inhibitor users and in 130 of 334 non-users.

**Figure 3:**
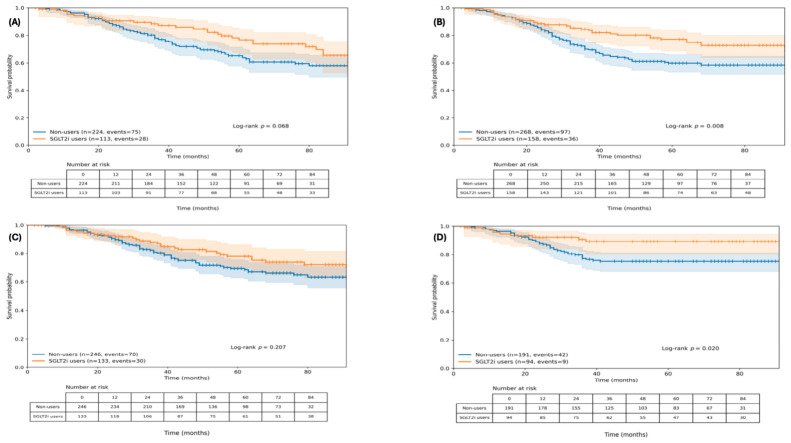
Kaplan–Meier Curves for Overall Survival According to SGLT2 Inhibitor Use in Clinically Relevant Subgroups. Log-rank *p* values were 0.068 in patients without diabetes mellitus (**A**), 0.008 in patients without heart failure (**B**), 0.207 in patients without chronic kidney disease (**C**), and 0.020 in patients without both heart failure and diabetes mellitus (**D**). Shaded areas represent 95% confidence intervals, tick marks indicate censored observations, and numbers at risk are shown below each panel. The endpoint was all-cause mortality. CKD: chronic kidney disease; DM: diabetes mellitus; HF: heart failure; SGLT2i: sodium–glucose cotransporter-2 inhibitor.

Exploratory subgroup analyses were performed according to diabetes mellitus, chronic kidney disease, heart failure, and the combined absence of heart failure and diabetes mellitus. Among patients with diabetes mellitus, all-cause mortality was lower in SGLT2 inhibitor users than in non-users (29.0% vs. 50.0%; *p* = 0.002). Among patients without diabetes mellitus, mortality was numerically lower in SGLT2 inhibitor users (24.8% vs. 33.5%; *p* = 0.102), and Kaplan–Meier analysis showed a nonsignificant trend toward higher survival probability (log-rank *p* = 0.068; Figure 3A).

Among patients without heart failure, SGLT2 inhibitor use was associated with lower all-cause mortality in bivariate analysis (22.8% vs. 36.2%; *p* = 0.005) and higher survival probability on Kaplan–Meier analysis (log-rank *p* = 0.008; Figure 3B). Among patients with heart failure, mortality was numerically lower in SGLT2 inhibitor users than in non-users (39.6% vs. 50.0%; *p* = 0.272).

Among patients with chronic kidney disease, all-cause mortality was lower in SGLT2 inhibitor users than in non-users (34.2% vs. 68.2%; *p* < 0.001). Among patients without chronic kidney disease, mortality was numerically lower in SGLT2 inhibitor users (22.6% vs. 28.5%; *p* = 0.214), and survival probability was numerically higher without a statistically significant between-group difference (log-rank *p* = 0.207; Figure 3C).

Among patients without both heart failure and diabetes mellitus, SGLT2 inhibitor use was associated with lower all-cause mortality in bivariate analysis (9.6% vs. 22.0%; *p* = 0.010) and higher survival probability on Kaplan–Meier analysis (log-rank *p* = 0.020; Figure 3D).

## 4. Discussion

In this cohort of 540 patients with preserved LVEF undergoing CIED implantation for cardiac conduction disease, documented SGLT2 inhibitor use within 3 months after implantation was independently associated with lower all-cause mortality (HR, 0.48; 95% CI, 0.38–0.69; *p* < 0.001), with higher survival probability also observed on Kaplan–Meier analysis. Despite preserved LVEF, all-cause mortality occurred in 34.3% of patients during a median follow-up of 64.2 months. Older age, complete LBBB, lower GFR, higher log-transformed NT-proBNP, and greater IVS thickness were also independently associated with mortality. Exploratory subgroup analyses showed significantly higher survival probability among SGLT2 inhibitor users without heart failure and among those without both heart failure and diabetes mellitus, whereas differences in patients without diabetes mellitus or chronic kidney disease did not reach statistical significance. Given the retrospective observational design, these findings indicate an association rather than a causal treatment effect and suggest that risk after CIED implantation reflects an integrated electrical, structural, renal, and neurohormonal substrate rather than LVEF alone.

Patients requiring CIED implantation for conduction disease are commonly older and have substantial cardiovascular and cardiorenal comorbidity. Although ventricular pacing burden was unavailable, the predominance of high-grade and complete AV block suggests that many patients were likely exposed to frequent ventricular pacing. In the PACE trial, patients with bradycardia and preserved systolic function who underwent right ventricular apical pacing developed progressive left ventricular remodeling and functional decline, whereas these adverse effects were less pronounced with biventricular pacing [1]. Current cardiac physiologic pacing recommendations therefore support periodic assessment of ventricular function in patients with chronic LBBB or a high anticipated burden of right ventricular pacing [20]. Observational evidence also indicates that an RV pacing burden of approximately 20–40% or greater may increase the risk of electrical dyssynchrony, pacing-induced cardiomyopathy, and heart failure, while biventricular or conduction system pacing may reduce this risk in appropriately selected patients [21,22,23,24]. However, because pacing burden, paced QRS duration, device interrogation data, and longitudinal echocardiographic measurements were unavailable in the present study, these mechanisms should be regarded as clinically plausible background rather than demonstrated mediators of the observed survival association.

The association between SGLT2 inhibitor use and survival is directionally consistent with the broader cardiorenal evidence base. Randomized trials have established SGLT2 inhibitors as effective therapies for reducing heart failure events across the spectrum of LVEF, and contemporary guidelines recommend these agents for symptomatic heart failure, including HFmrEF and HFpEF [3,4,5,6,7,25]. More broadly, pooled evidence from 15 major randomized trials demonstrated that SGLT2 inhibitors were associated with lower risks of heart failure events and cardiovascular mortality across diverse populations, including patients with heart failure, type 2 diabetes, chronic kidney disease, or atherosclerotic cardiovascular disease [26]. These data do not establish a treatment effect in patients undergoing CIED implantation for conduction disease. Rather, they provide a biologically and clinically relevant context for the association observed in our cohort, whose defining characteristic was device-treated conduction disease rather than symptomatic heart failure.

When baseline characteristics were evaluated according to SGLT2 inhibitor use, these agents were more frequently prescribed to patients with comorbidities such as diabetes mellitus, coronary artery disease, hypertension, and chronic kidney disease. Although SGLT2 inhibitor use was also numerically more common among patients with heart failure, the difference did not reach statistical significance. Interestingly, a history of syncope or presyncope was more frequent among SGLT2 inhibitor users. As no direct evidence in the literature clearly explains this association, we considered it likely to be an incidental finding related to the greater comorbidity burden and concomitant medical treatment in these patients. The higher use of SGLT2 inhibitors across comorbidity groups other than heart failure may also reflect the chronological expansion of their clinical indications. The initial demonstration of cardiovascular benefits [27,28]., followed by evidence supporting favorable renal effects [29,30] and, subsequently, benefits across the full spectrum of heart failure [25,31] may have contributed to the observed prescribing patterns in our cohort.

The most directly relevant external evidence is emerging from device-treated populations. In a recent population-based study of 11,518 eligible conventional pacemaker recipients with atrioventricular block, Avidan et al. performed propensity-score matching and generated two well-balanced cohorts of 1226 SGLT2 inhibitor users and 1226 non-users. Over 3 years, all-cause mortality occurred in 10.1% of SGLT2 inhibitor users versus 15.8% of non-users (HR, 0.62; 95% CI, 0.50–0.78; *p* < 0.001), while heart failure hospitalization occurred in 10.7% versus 22.8%, respectively (HR, 0.50; 95% CI, 0.41–0.62; *p* < 0.001). Events occurring during the first 3 months after implantation were excluded to reduce peri-procedural confounding [14]. Younis et al. evaluated 13,888 consecutive CIED recipients from two tertiary centers, including 696 patients exposed to SGLT2 inhibitors, with treatment modeled as a time-dependent variable. SGLT2 inhibitor use was associated with a 22% lower risk of atrial tachyarrhythmias (HR, 0.78; 95% CI, 0.70–0.87; *p* < 0.001) and a 35% lower risk of all-cause mortality (HR, 0.65; 95% CI, 0.45–0.92; *p* = 0.015), without a significant reduction in ventricular tachyarrhythmias (HR, 0.92; 95% CI, 0.80–1.06; *p* = 0.26) [13]. Although these studies differed from ours in population, exposure definition, endpoints, and analytical approach, their results support prospective evaluation of SGLT2 inhibitors in device-treated populations. Our study adds data from a cohort defined by preserved LVEF and conduction disease, including AV block, non-AV conduction disease, and AF with a slow ventricular response.

The subgroup analyses provide an important but exploratory perspective. SGLT2 inhibitor use was associated with lower all-cause mortality among patients with diabetes mellitus and chronic kidney disease, consistent with evidence from large-scale studies [28,30]. Among patients with heart failure, mortality was numerically lower among SGLT2 inhibitor users but did not reach statistical significance, possibly because of the small subgroup size and limited number of events. Furthermore, the relatively low prevalence of heart failure in our cohort reduces the likelihood that the overall association between SGLT2 inhibitor use and mortality was driven primarily by the established benefits of these agents in patients with heart failure. Conversely, had heart failure been more prevalent, treatment selection based on this conventional indication could have exerted a greater influence on the observed association and complicated the interpretation of our primary hypothesis.

In patients without heart failure, SGLT2 inhibitor use was associated with lower all-cause mortality in bivariate analysis and higher survival probability on Kaplan–Meier analysis. A similar pattern was observed among patients without both heart failure and diabetes mellitus. These observations lessen, but do not eliminate, the possibility that the overall association was driven solely by conventional SGLT2 inhibitor indications. In contrast, the mortality and survival differences in patients without diabetes or without chronic kidney disease were directionally similar but did not reach statistical significance, which may reflect smaller subgroup sizes and fewer events. The Kaplan–Meier curves in the subgroup without both heart failure and diabetes mellitus should be interpreted carefully. The curves were initially close, separated during early-to-intermediate follow-up, and then remained broadly parallel. This pattern may reflect an early difference in event accumulation, but it cannot establish an early treatment effect, a time-varying hazard ratio, or sustained benefit. The later parallel course may be influenced by fewer patients remaining at risk, fewer late events, residual baseline imbalance, or treatment-selection effects. Moreover, these subgroup analyses were unadjusted, no subgroup-specific multivariable Cox models or formal interaction tests were performed, and the log-rank test assesses the overall difference across follow-up rather than the timing of a difference. This finding should therefore be regarded as a hypothesis-generating survival signal. The need for such caution is underscored by DAPA-MI, in which dapagliflozin improved a hierarchical cardiometabolic outcome in patients with myocardial infarction without diabetes or chronic heart failure but did not reduce the conventional composite of cardiovascular death or heart failure hospitalization [32]. Consequently, CIED implantation alone should not be considered an indication for SGLT2 inhibitor treatment.

Several mechanisms could plausibly contribute to the observed association, although none was directly assessed in the present study. SGLT2 inhibitors may improve cardiorenal physiology through natriuresis, reduced intraglomerular pressure, attenuation of congestion, and favorable effects on myocardial energetics and loading conditions [10]. Cardiac magnetic resonance meta-analyses have suggested favorable changes in left ventricular volumes and myocardial mass with SGLT2 inhibitor treatment, while an updated meta-analysis reported improved right ventricular performance in heart failure populations [11,26]. Meta-analytic data have also suggested lower risks of atrial arrhythmias and sudden cardiac death with SGLT2 inhibitors in selected populations with heart failure or diabetes mellitus [8,9]. Consistent with these observations, prospective device-monitoring data have reported lower device-detected atrial tachyarrhythmia burden among SGLT2 inhibitor users [33]. Additional ICD-based observational studies and meta-analytic analyses have suggested possible reductions in ventricular arrhythmias, appropriate ICD therapies, and sudden cardiac death; however, the magnitude and consistency of these associations appear to vary according to the study population, baseline heart failure status, device type, and endpoint definition [34,35,36]. Such pathways may be particularly relevant in patients with conduction disease and likely pacing exposure, in whom electrical dyssynchrony, structural remodeling, elevated filling pressures, and atrial arrhythmogenicity may coexist. Nevertheless, the present study cannot determine whether the observed mortality association was mediated by reverse remodeling, renal protection, reduced congestion, attenuation of arrhythmic burden, or other unmeasured mechanisms.

The additional prognostic findings are clinically coherent. Older age likely reflects frailty, multimorbidity, and competing risks; complete LBBB may indicate a more advanced electrical and structural myocardial substrate; and lower GFR and higher NT-proBNP identify cardiorenal vulnerability and hemodynamic stress. Greater IVS thickness may reflect hypertensive remodeling, myocardial hypertrophy, or other adverse structural substrates. These variables should be interpreted as risk markers rather than causal pathways. Although heart failure and AF were more prevalent among patients who died, neither remained independently significant after adjustment, possibly because their associations overlapped with age, renal dysfunction, biomarker burden, and structural myocardial disease. Clinically, preserved LVEF should not be considered sufficient evidence of favorable long-term prognosis after CIED implantation for conduction disease. Risk assessment should incorporate age, renal function, biomarkers of hemodynamic stress, electrical substrate, structural remodeling, and comorbidity burden. The present findings do not support routine initiation of SGLT2 inhibitors solely because a patient receives a CIED; however, they support systematic evaluation for guideline-based indications and prospective investigation of early SGLT2 inhibitor strategies in high-risk device-treated patients with preserved LVEF.

This study included a relatively large, well-characterized cohort of patients with preserved LVEF undergoing CIED implantation specifically for cardiac conduction disease, an understudied but clinically common population. The median follow-up of approximately 64 months and ascertainment of mortality through institutional records and a national death registry enabled comprehensive evaluation of all-cause mortality. The multivariable model incorporated clinical, electrocardiographic, echocardiographic, renal, hematologic, and biomarker variables, with no evidence of substantial multicollinearity. In addition, overall and subgroup survival analyses provided complementary information on the consistency of the observed association between early documented SGLT2 inhibitor use and survival.

The observational, retrospective, and single-center nature of the study limits causal interpretation and cannot eliminate residual confounding, selection bias, or confounding related to treatment allocation. A major limitation of the study was the incomplete characterization of SGLT2 inhibitor exposure. Detailed information regarding treatment indication, agent, dose, adherence, persistence, discontinuation, and changes in therapy during follow-up was not consistently available from the national prescription database or institutional records. The absence of these longitudinal treatment data may have resulted in exposure misclassification and may have influenced the observed association between SGLT2 inhibitor use and mortality. Detailed device-related data, including ventricular pacing burden, paced QRS duration, conduction system pacing status, device interrogation findings, and arrhythmia burden, as well as serial echocardiographic measurements, were unavailable. Consequently, pacing-related remodeling and potential mechanistic mediation could not be assessed. Despite adjustment for the available baseline clinical variables in the multivariable Cox regression model, confounding by indication and residual baseline risk imbalance between SGLT2 inhibitor users and non-users cannot be excluded. Propensity-score matching, inverse probability weighting, and standardized mean difference analyses were not performed. Therefore, the multivariable analysis may not have fully accounted for measured and unmeasured differences between the treatment groups. All-cause mortality was the sole endpoint, and cause-specific death, cardiovascular mortality, heart failure hospitalization, device therapies, and arrhythmia-related outcomes were not evaluated. Finally, subgroup analyses were exploratory and unadjusted, without formal interaction testing or subgroup-specific Cox models, and the older, comorbid single-center population may limit generalizability to lower-risk populations and contemporary centers with broader use of physiologic pacing strategies.

## 5. Conclusions

In patients with preserved LVEF undergoing CIED implantation for cardiac conduction disease, documented SGLT2 inhibitor use within 3 months after implantation was independently associated with lower all-cause mortality and higher survival probability during follow-up. However, these findings are hypothesis-generating and should not be interpreted as demonstrating a causal therapeutic benefit. The overall Kaplan–Meier analysis and exploratory subgroup findings, including the subgroup without both heart failure and diabetes mellitus, support further investigation but do not establish causality. Prospective multicenter studies using time-varying exposure assessment, detailed device interrogation and pacing data, longitudinal echocardiography, and adjudicated cardiovascular outcomes are needed to clarify whether SGLT2 inhibitors provide prognostic benefit in device-treated conduction disease populations.

## Figures and Tables

**Table 1:** Clinical and Demographic Characteristics According to All-Cause Mortality.

	All-Cause Mortality (−) (n: 355)	All-Cause Mortality (+) (n: 185)	*p* Value
Age, (years) *	76 (24–97)	86 (60–102)	**<0.001**
Sex, n (%)			
● Female Sex	195 (54.9)	97 (52.4)	0.581
Indications for Implantation, n (%)			
● AV Block ^#^	224 (63.1)	116 (63.7)	
● Non-AV conduction disease ^##^	43 (12.1)	19 (10.3)	0.770
● AF with slow ventricular response	88 (24.8)	50 (27.0)	
Types of Implanted CIEDs, n (%)			
● VR pacemaker	101 (28.5)	81 (43.8)	
● DR pacemaker	214 (60.3)	82 (44.3)	**<0.001**
● VR-ICD	20 (5.6)	18 (9.7)	
● DR-ICD	20 (5.6)	4 (2.2)	
Heart Failure, n (%)			
● Patients without HF	293 (82.5)	133 (71.9)	**0.006**
● Patients with HF and LVEF ≥ 50%	62 (17.5)	52 (28.1)	
DM, n (%)	121 (34.1)	82 (44.3)	**0.020**
HT, n (%)	285 (80.3)	166 (89.2)	**0.005**
Stroke, n (%)	83 (23.4)	61 (33.0)	**0.017**
CAD **, n (%)	91 (25.6)	59 (31.9)	0.123
CKD ***, n (%)	76 (21.4)	85 (45.9)	**<0.001**
AF, n (%)	147 (41.4)	80 (43.2)	0.594
Syncope-presyncope, n (%)	275 (77.5)	128 (69.2)	**0.016**
Smoker, n (%)	68 (19.2)	28 (15.1)	0.246
Medical therapy; n (%)			
● Beta Blocker	244 (68.7)	139 (75.1)	0.121
● Non-DHP Calcium Channel Blocker	65 (18.3)	47 (25.4)	0.054
● Amiodarone	20 (5.6)	21 (11.4)	**0.017**
● Class 1 Antiarrhythmic Drugs	11 (3.0)	7 (3.8)	0.715
● Digoxin	65 (18.3)	49 (26.5)	**0.027**
● ACE-i/ARB	300 (84.5)	163 (88.1)	0.256
● SGLT2 inhibitor	151 (42.5)	55 (29.7)	**0.004**
● ARNI	8 (2.3)	4 (2.2)	0.946
● MRA	96 (27.0)	91 (49.2)	**<0.001**
● Diuretic	285 (80.3)	178 (90.2)	**<0.001**
● Oral Anticoagulant Drugs	249 (70.1)	152 (82.2)	**0.002**

ACE-i: angiotensin-converting enzyme inhibitor; ARB: angiotensin receptor blocker; AF: atrial fibrillation; ARNI: angiotensin receptor-neprilysin inhibitor; AV: atrioventricular; CAD: coronary artery disease; CIED: cardiac implantable electronic device; CKD: chronic kidney disease; DM: diabetes mellitus; DR: dual-chamber; HF: heart failure; HT: hypertension; ICD: implantable cardioverter-defibrillator; MRA: mineralocorticoid receptor antagonist; Non-DHP: non-dihydropyridine; SGLT2: sodium–glucose cotransporter-2; VR: ventricular single-chamber. * Median (minimum–maximum). ** CAD was defined as ≥50% coronary stenosis on invasive coronary angiography and/or a history of percutaneous coronary intervention or coronary artery bypass grafting. *** CKD was defined as an estimated glomerular filtration rate < 60 mL/min/1.73 m^2^. ^#^ AV block included 2:1 AV block causing symptomatic bradycardia, high-grade AV block, and complete AV block. ^##^ Non-AV conduction disease included bifascicular or trifascicular conduction disease with symptomatic bradycardia. *p* values presented in bold indicate statistically significant results.

**Table 2:** Clinical and Demographic Characteristics According to SGLT2 Inhibitor Use.

	SGLT2 Inhibitor Non-Users (−) (n: 334)	SGLT2 Inhibitor Users (+) (n: 206)	*p* Value
Age, (year) *	79 (39–98)	79 (51–102)	0.271
Sex, n (%)			
● Female Sex	179 (53.6)	113 (54.9)	0.775
Indications for Implantation, n (%)			
● AV Block ^#^	214 (64.1)	126 (61.2)	
● Non-AV conduction disease ^##^	36 (10.8)	26 (12.6)	0.739
● AF with slow ventricular response	84 (25.1)	54 (26.2)	
Types of Implanted CIEDs, n (%)			
● VR pacemaker	121 (36.2)	61 (29.6)	
● DR pacemaker	172 (51.5)	124 (60.2)	0.179
● VR-ICD	27 (8.1)	11 (5.3)	
● DR-ICD	14 (4.2)	10 (4.9)	
Heart Failure; n (%)			
● Patients without HF	268 (80.2)	158 (76.7)	0.327
● Patients with HF and LVEF ≥ 50%	66 (19.8)	48 (23.3)	
DM, n (%)	110 (32.9)	93 (45.1)	**0.004**
HT, n (%)	267 (79.9)	184 (89.3)	**0.004**
Stroke, n (%)	88 (26.3)	56 (27.2)	0.831
CAD **, n (%)	75 (22.5)	75 (36.2)	**<0.001**
CKD ***, n (%)	88 (26.3)	73 (35.4)	**0.022**
AF, n (%)	132 (39.5)	95 (46.1)	0.132
Syncope-presyncope, n (%)	238 (71.3)	165 (80.1)	**0.025**
Smoker, n (%)	61 (18.3)	35 (17.1)	0.707

AF: atrial fibrillation; AV: atrioventricular; CAD: coronary artery disease; CIED: cardiac implantable electronic device; CKD: chronic kidney disease; DM: diabetes mellitus; DR: dual-chamber; HF: heart failure; HT: hypertension; ICD: implantable cardioverter-defibrillator; SGLT2: sodium–glucose cotransporter-2; VR: ventricular single-chamber. * Median (minimum–maximum). ** CAD was defined as ≥50% coronary stenosis on invasive coronary angiography and/or a history of percutaneous coronary intervention or coronary artery bypass grafting. *** CKD was defined as an estimated glomerular filtration rate < 60 mL/min/1.73 m^2^. ^#^ AV block included 2:1 AV block causing symptomatic bradycardia, high-grade AV block, and complete AV block. ^##^ Non-AV conduction disease included bifascicular or trifascicular conduction disease with symptomatic bradycardia. *p* values presented in bold indicate statistically significant results.

**Table 3:** Electrocardiographic and Echocardiographic Characteristics According to All-cause Mortality.

	All-Cause Mortality (−) (n = 355)	All-Cause Mortality (+) (n = 185)	*p* Value
PR interval, ms	200 (106–400)	210 (138–340)	0.484
QRS duration, ms	132 (65–240)	140 (81–180)	**0.001**
QTc interval, ms	435 (328–536)	440 (370–535)	**0.018**
Complete AV block, n (%)	115 (32.4)	70 (37.7)	0.092
High-degree AV block ^#^, n (%)	54 (15.2)	32 (17.3)	0.149
LVEF, % *	60 (50–65)	60 (50–64)	0.389
LA, mm *	44 (29–60)	45 (32–62)	**0.001**
LVEDd, mm *	50 (37–59)	51 (36–60)	0.169
IVS, mm *	11 (7–18)	12 (8–20)	**0.009**
PW, mm *	11 (6–17)	11 (8–19)	0.062
TAPSE, mm *	20 (10–27)	20 (14–28)	0.349
sPAP, mmHg *	35 (20–100)	38 (22–100)	**0.003**

AV: atrioventricular; IVS: interventricular septum; LA: left atrium; LVEF: left ventricular ejection fraction; LVEDd: left ventricular end-diastolic diameter; PW: posterior wall thickness; sPAP: systolic pulmonary artery pressure; TAPSE: tricuspid annular plane systolic excursion. * Median (minimum–maximum). ^#^ High-degree AV block included AV block greater than 2:1 without complete AV block on the baseline ECG. *p* values presented in bold indicate statistically significant results.

**Table 4:** Laboratory and Biochemical Characteristics According to All-cause Mortality.

	All-Cause Mortality (−) (n = 355)	All-Cause Mortality (+) (n = 185)	*p* Value
Hemoglobin, g/dL *	13.2 (6.5–19.0)	12.2 (5.2–17.0)	**<0.001**
Hematocrit, % *	38.3 (19.6–56.0)	36.3 (16.2–50.0)	**<0.001**
Platelet count, ×10^3^/µL *	221.0 (50.2–535.0)	211.5 (42.0–676.0)	0.067
White blood cell count, ×10^3^/µL *	7.2 (3.2–14.7)	7.6 (2.3–15.6)	0.179
Neutrophil count, ×10^3^/µL *	4.3 (0.3–11.4)	4.8 (1.5–10.8)	**0.001**
Lymphocyte count, ×10^3^/µL *	1.8 (0.3–4.5)	1.5 (0.2–4.2)	**<0.001**
Monocyte count, ×10^3^/µL *	0.6 (0.1–2.0)	0.6 (0.1–1.3)	**0.012**
Creatinine, mg/dL *	0.9 (0.5–5.6)	1.1 (0.5–8.8)	**<0.001**
GFR, mL/min/1.73 m^2^ *	67.1 (12.0–128.2)	51.5 (8.2–120.0)	**<0.001**
Sodium, mmol/L *	140.1 (127.5–152.0)	141.0 (126.3–147.7)	0.898
Potassium, mmol/L *	4.5 (3.3–5.7)	4.5 (3.0–5.9)	0.974
LDL cholesterol, mg/dL *	108.0 (15.0–251.0)	112.0 (46.0–216.0)	0.315
Troponin T, ng/mL *	0.012 (0.001–0.240)	0.016 (0.001–0.330)	**0.001**
NT-proBNP, pg/mL *	167.7 (3.5–2389.4)	275.0 (56.5–3277.0)	**0.001**
CRP, mg/dL *	3.9 (0.1–75.0)	7.8 (0.3–86.0)	**<0.001**

CRP: C-reactive protein; GFR: glomerular filtration rate; Hb: hemoglobin; HCT: hematocrit; LDL: low-density lipoprotein; NEUT: neutrophil; NT-proBNP: N-terminal pro-B-type natriuretic peptide; PLT: platelet; WBC: white blood cell. * Median (minimum–maximum). *p* values presented in bold indicate statistically significant results.

**Table 5:** Multivariable Cox Regression Analysis for All-cause Mortality.

	Hazard Ratio	95% Confidence Interval	*p* Value
Age, per year	1.05	1.03–1.07	**<0.001**
Male sex	1.10	0.79–1.55	0.571
Heart failure	0.91	0.66–1.27	0.564
Coronary artery disease	1.34	0.96–1.81	0.087
Diabetes mellitus	1.09	0.80–1.50	0.561
Hypertension	0.65	0.38–1.09	0.110
Prior stroke	1.10	0.79–1.54	0.573
Atrial fibrillation	1.41	0.99–1.91	0.061
Complete LBBB	1.57	1.29–2.24	**0.001**
Complete AV block	1.32	0.97–1.80	0.077
LVEDd, per mm	1.00	0.97–1.08	0.669
LVEF, per %	0.98	0.93–1.02	0.332
IVS, per mm	1.18	1.03–1.42	**0.022**
Hemoglobin, per g/dL	0.94	0.86–1.03	0.229
White blood cell count, per 10^3^/µL	1.00	0.99–1.01	0.151
GFR, per mL/min	0.98	0.97–0.99	**0.004**
Troponin, per ng/mL	0.48	0.04–1.42	0.074
Log-transformed NT-proBNP	1.26	1.12–1.41	**0.003**
Beta-blocker use	1.14	0.78–1.65	0.488
ACE-i/ARB use	1.01	0.62–1.64	0.957
SGLT2 inhibitor use	0.48	0.38–0.69	**<0.001**

SGLT2 inhibitor use: Documented active use or treatment initiation within 3 months after CIED implantation. ACE-i: angiotensin-converting enzyme inhibitor, ARB: angiotensin receptor blocker, AF: atrial fibrillation, AV: atrioventricular, CKD: chronic kidney disease, CRP: c reactive protein, DM: diabetes mellitus, GFR: glomerular filtration rate, Hb: haemoglobin, HF: heart failure, HT: hypertension, IVS: interventricular septum, LDL: low density lipoprotein, LVEDd: left ventricular end-diastolic diameter, NT-pro BNP: N-terminal protein brain natriuretic peptide, SGLT2: sodium–glucose cotransporter-2, sPAP: systolic pulmonary arterial pressure, TAPSE: tricuspid annular plane systolic excursion, WBC: white blood cells. *p* values presented in bold indicate statistically significant results.

## Data Availability

The data presented in this study are available on request from the corresponding author due to privacy considerations and institutional restrictions related to potentially identifiable clinical information. The data are not publicly available.
